# MOMC-2. Glioblastoma Metabolic Symbiosis: When Lactate Takes The Lead

**DOI:** 10.1093/noajnl/vdab070.012

**Published:** 2021-07-05

**Authors:** Joris Guyon, Claire Larrieu, Cyrielle Bouchez, Ignacio Fernandez Moncada, Aurelien Coffe, Macha Nikolski, Andreas Bikfalvi, Thomas Daubon

**Affiliations:** 1 INSERM U1029, Bordeaux, France; 2 CNRS UMR5095, Bordeaux, France

## Abstract

Glioblastoma (GBM) is a common and devastating brain tumor, associated with a low median survival, despite standard therapeutic management. Among its major features, GBMs are highly angiogenic and exhibit paradoxically an elevated glycolysis. Most of differentiated cells convert glucose into pyruvate that enters into the Krebs cycle to maximize energy production in the presence of oxygen. For cancer cells, glucose uptake and catabolism are increased regardless of oxygen level. However, their energy needs are important – mainly for rapid growth – that it requires a much faster production flow. It is at this step that lactate dehydrogenase (LDH) are involved: LDHA converts efficiently pyruvate into lactate and generates NAD^+^ to maintain glycolysis. Thus, the lactate formed is exported into the extracellular compartment inducing an unfavourable acidification of the microenvironment. Moreover, LDHB, another LDH isoform, metabolizes lactate into pyruvate for generating energy in mitochondria. Though LDHA has already been studied in many cancers including GBM, the simultaneous role of LDH enzymes have not yet been investigated in GBM development.

Hypoxia-driven LDHA expression and lactate production increased cell invasion. Infusing 13C-lactate in starved cells rescued TCA cycle. Then, we showed that, under hypoxia, double sgLDHA/B cell growth and invasion was dramatically decreased in comparison to control cells, mainly caused by an increase in apoptosis. Moreover, double impairment of LDHA and B significantly reduced tumor growth and cell invasion, and induces a massive increase in mouse survival. Tracing experiments with 13C-Glucose coupled with RNA sequencing revealed how metabolism adapts to these contraints, by modifying electron transport chain subunit expressions or by increasing lipid droplet formation.

Considered for a long time as a metabolic waste, lactate is shown here to play a critical role in GBM cell symbiosis. This study highlighted GBM adaptability through the LDH isoforms and their involvement in GBM development.

